# A Systematic Review on the Impact of Mental Health First Aid in Medical, Nursing and Allied Healthcare Professional Students

**DOI:** 10.1192/bjo.2025.10322

**Published:** 2025-06-20

**Authors:** Su Ying Yeoh, Joanne Rodda, Sharna Bennett, Helen Bintley

**Affiliations:** 1 Kent and Medway NHS and Social Care Partnership Trust, Kent, United Kingdom; 2 Kent and Medway Medical School, Canterbury, United Kingdom

## Abstract

**Aims:** The unique demands of studying for a healthcare-related degree are associated with an increased risk of developing mental health problems. Stigma and fear of repercussions for their future careers may be a barrier to these students seeking professional help. Mental Health First Aid (MHFA) is a globally disseminated psycho-education course, training members of the public to recognise and respond to people experiencing mental health problems in their communities. Training healthcare professional (HCP) students in MHFA may help them support their peers and optimise their own wellbeing.

We aimed to review the literature regarding experiences of MHFA in medical, nursing and allied HCP students and its impact on mental health knowledge, confidence and skills in supporting others, stigmatising attitudes, self-care, peer support and student wellbeing.

**Methods:** This systematic review was conducted in accordance with PRISMA guidelines and registered on PROSPERO (ID: CRD42024589509). Electronic databases (Ovid, Medline, PsycINFO, CINAHL and ERIC) were searched on 9/10/2024 for primary studies evaluating the impact of MHFA in HCP students. Data was extracted independently by two authors. Study quality was assessed using the MMERSQI (Modified Medical Education Research Study Quality Instrument) and Cochrane Risk of Bias tool.

**Results:** Searches identified 1613 studies, 25 of which met inclusion criteria. Study types were heterogeneous, including four randomised controlled trials (RCTs) and four qualitative studies. Eight studies reported that MHFA significantly improved post-test knowledge and mental health literacy. Fourteen studies reported enhanced confidence in providing MHFA post-training. Three studies described outcomes based on performance on simulation assessments, and eight studies reported reduction in discriminatory attitudes following MHFA training. Data on peer support and student wellbeing was limited. Qualitative studies suggested that MHFA clarified misconceptions around mental illness, facilitated supporting friends and family, and helped with self-care and seeking professional support for their own mental health. Student pharmacists felt that skills gained from MHFA were particularly advantageous in their professional roles. Qualitative data also suggested that tailored content for HCP students and increased pace of delivery would be beneficial.

**Conclusion:** The evidence base on this topic is limited, with many small studies and few RCTs. However, the available literature suggests that MHFA may be a promising strategy to improve mental health literacy, confidence, skills, and reduce stigma in HCP students. Findings suggest that there is demand for bespoke MHFA courses for HCP students, which may fulfil an unmet need in facilitating them to provide support to their peers, and optimise their own wellbeing.

